# Meta‐Analysis of the Safety and Efficacy of Intensive Blood Pressure Control After Thrombectomy

**DOI:** 10.1002/brb3.70211

**Published:** 2025-02-09

**Authors:** Qiang Ji Bao, Yi Ming Li, Xinting Wu, Yun Ting Li, Xiao Long Huang, Hui Zhou, Xiao Qiang Zhang, Xue Jun Wang

**Affiliations:** ^1^ Department of Neurosurgery Guang'an People's Hospital Guang'an Sichuan China; ^2^ Department of Clinical Medicine, Graduate School Qinghai University Xining Qinghai China; ^3^ Department of Anesthesia Guang'an People's Hospital Guang'an Sichuan China; ^4^ Department of Anesthesia Red Cross Hospital in Qinghai Province Xining Qinghai China

**Keywords:** blood pressure, endovascular thrombectomy, ischemic stroke, meta‐analysis, outcomes

## Abstract

**Purpose:**

After successful endovascular treatment of acute ischemic stroke, there remains considerable controversy surrounding the efficacy of intensified blood pressure control therapy. Presently, numerous randomized controlled trials have yielded diverse findings. Thus, our objective is to consolidate all current randomized controlled trial data to evaluate whether intensified systolic blood pressure targets, in comparison to standard targets, offer superior safety and efficacy.

**Methods:**

By searching the EMBASE, PubMed, and Cochrane Library databases, we identified randomized controlled trials comparing standard blood pressure control to intensified blood pressure control in patients with acute ischemic stroke undergoing endovascular thrombectomy (EVT). Efficacy outcomes included favorable clinical outcomes (defined as a modified Rankin Scale (mRS) score of 0–2 at 90 days), excellence clinical outcomes (defined as an mRS score of 0–1 at 90 days), and 90‐day mortality. Safety outcomes included symptomatic intracranial hemorrhage (sICH). The relationship between standard and intensified blood pressure control post‐EVT and the prognosis of patients with acute ischemic stroke undergoing endovascular thrombectomy was expressed using risk ratios (RR) and their corresponding 95% confidence intervals (95% CI).

**Results:**

The analysis encompassed four studies involving a total of 753 patients. After sensitivity analysis and exclusion of literature with significant heterogeneity, it was revealed that compared to intensified blood pressure control, standard blood pressure control was associated with excellent clinical outcomes RR of 0.81 (95% confidence interval [CI]: 0.73–0.90; *p* < 0.05; *I* ^2^  =  25%). However, no significant correlation was found for favorable clinical outcomes, 90‐day mortality, and safety outcomes including symptomatic intracranial hemorrhage.

**Conclusions:**

After careful analysis, our conclusion is that intensified blood pressure control, compared to standard blood pressure control following endovascular treatment in acute stroke patients, does not yield better clinical outcomes and may even lead to inferior ones. Moreover, there is no significant disparity in terms of safety between the two approaches.

## Graphical abstract

Comparing intensive systolic blood pressure (SBP) target with standard SBP target on the study outcomes. (A) The favorable clinical outcome (mRS ≤ 2) at 90 days. Intensive SBP target significantly improved the favorable clinical outcome at 90 days (mRS ≤ 2), heterogeneity: *I* ^2^ = 15%; *p* = 0.32. (B) The excellent clinical outcome (mRS ≤ 1) at 90 days. At 90 days, the intensive SBP target significantly increased the rate of excellent clinical outcomes (mRS ≤ 1), heterogeneity: *I* ^2^ = 25%; *p* = 0.26. (C) The mortality at 90 days. There was no significant difference in mortality at 90 days between the intensive and standard SBP targets, heterogeneity: *I* ^2^ = 0%; *p* = 0.53. (D) The occurrence of sICH at 90 days. The occurrence of symptomatic intracranial hemorrhage (sICH) at 90 days was similar between the intensive and standard SBP target groups, heterogeneity: *I* ^2^ = 0%; *p* = 0.72.

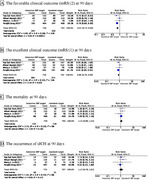

## 1 Introduction

Acute ischemic stroke represents a significant global health challenge, and endovascular thrombectomy (EVT) has emerged as a pivotal milestone in its treatment, profoundly altering the organization and operation of stroke services (Lapergue et al. 2017; Goyal et al. 2016). By eliminating obstruction or clots within arteries, EVT can effectively restore blood flow to ischemic regions in the brain, known as penumbral tissue (Lin et al. 2022; Wang et al. 2022). However, despite achieving favorable radiological outcomes, many patients exhibit poor functional recovery, with high risks of symptomatic intracranial hemorrhage and other forms of reperfusion injury. Consequently, there is a growing interest in adjunctive approaches post‐EVT to protect or sustain penumbral tissue from reperfusion injury. Blood pressure emerges as a modifiable factor to prevent reperfusion injury, given its frequent elevation and clear prognostic significance in acute ischemic stroke (Anadani et al. 2019; Mistry et al. 2018; Maïer et al. 2020; Samuels et al. 2021; Anadani et al. 2020). While guidelines advocate for conservative blood pressure control pre‐EVT and post‐EVT (Powers et al. 2019), recent confidence in EVT's efficacy, desire to mitigate ischemia‐reperfusion injury risks, and influential data linking presentation blood pressure to subsequent clinical outcomes have shifted opinions toward more aggressive blood pressure control in research and practice (Mulder et al. 2017). Nevertheless, in the absence of randomized evidence, guidelines continue to recommend maintaining lower blood pressure levels post‐EVT, consistent with those for patients eligible for intravenous thrombolysis after acute ischemic stroke (Powers et al. 2019; Turc et al. 2019). Hence, our aim is to assess the safety and efficacy of intensive blood pressure management compared to less intensive treatment in patients with successful reperfusion following EVT, addressing this controversy.

## 2 Methods and Analysis

We conducted and reported this systematic review in accordance with the Preferred Reporting Items for Systematic Reviews and Meta‐Analyses statement. The review protocol was registered in the International Platform of Registered Systematic Review and Meta‐analysis Protocols (INPLASY, https://inplasy.com/) with the registration number of INPLASY202460008.

### 2.1 Search Strategy

Conduct systematic reviews and meta‐analyses of RCTs according to the guidelines for systematic reviews and meta‐analyses (Page et al. 2021). Search the published literature in the EMBASE, PubMed, and Cochrane Library databases, primarily using search terms such as “ischemic stroke,” “endovascular thrombectomy,” and “blood pressure” supplemented with relevant synonyms to avoid missing literature during the search. The search terms will be provided in detail as online supplementary material (Search strategy).

### 2.2 Inclusion and Exclusion Criteria

After excluding duplicate samples, we screened the remaining literature according to the following criteria: (1) Cases of stroke diagnosed by imaging examinations. (2) Prospective studies comparing two different reinforcement standards for blood pressure treatment to assess their effects on stroke. (3) Studies with a sample size larger than 50 cases. We excluded conference abstracts, case reports, reviews, and letters from the search results of RCTs. Additionally, we excluded non‐English literature, abstracts, and studies unrelated to stroke. We assessed duplicate or overlapping data in publications and only included the most comprehensive studies. Unpublished data were not sought.

### 2.3 Data Extraction

Two independent researchers (BQJ, LYM) served as secondary evaluators, responsible for data extraction and cross‐verification. For the eligible studies, the following data were collected: basic information (including the first author's name, year of publication, and study region); patient characteristics (including the number of patients, mean age, medications prior to intervention, and medical history); and efficacy indicators (including favorable clinical outcomes with a mRS of 0–2, excellence clinical outcomes with a mRS of 0–1, and mortality rate). Any uncertainties or discrepancies were resolved through discussions between the researchers and the secondary evaluators to reach a consensus.

### 2.4 Study Quality Assessment and Statistical Analysis

We used the Newcastle–Ottawa Scale to assess the quality of these RCTs (Ottawa Hospital Research Institute [Internet] 2024).The heterogeneity was assessed using the I2 statistic and the chi‐square test. Heterogeneity was considered significant when I2 > 50%. If the included studies had I2 < 50% for the intervention outcomes, the fixed‐effect model of Mantel–Haenszel method was used. Otherwise, the random‐effects model of Mantel–Haenszel was employed. Visual funnel plots were used to evaluate publication bias (Figures S1–S4). The statistical significance was set at p‐value < 0.05, indicating a statistically significant result. All analyses were conducted using Review Manager (RevMan, version 5.4).

## 3 Results

**FIGURE 1:**
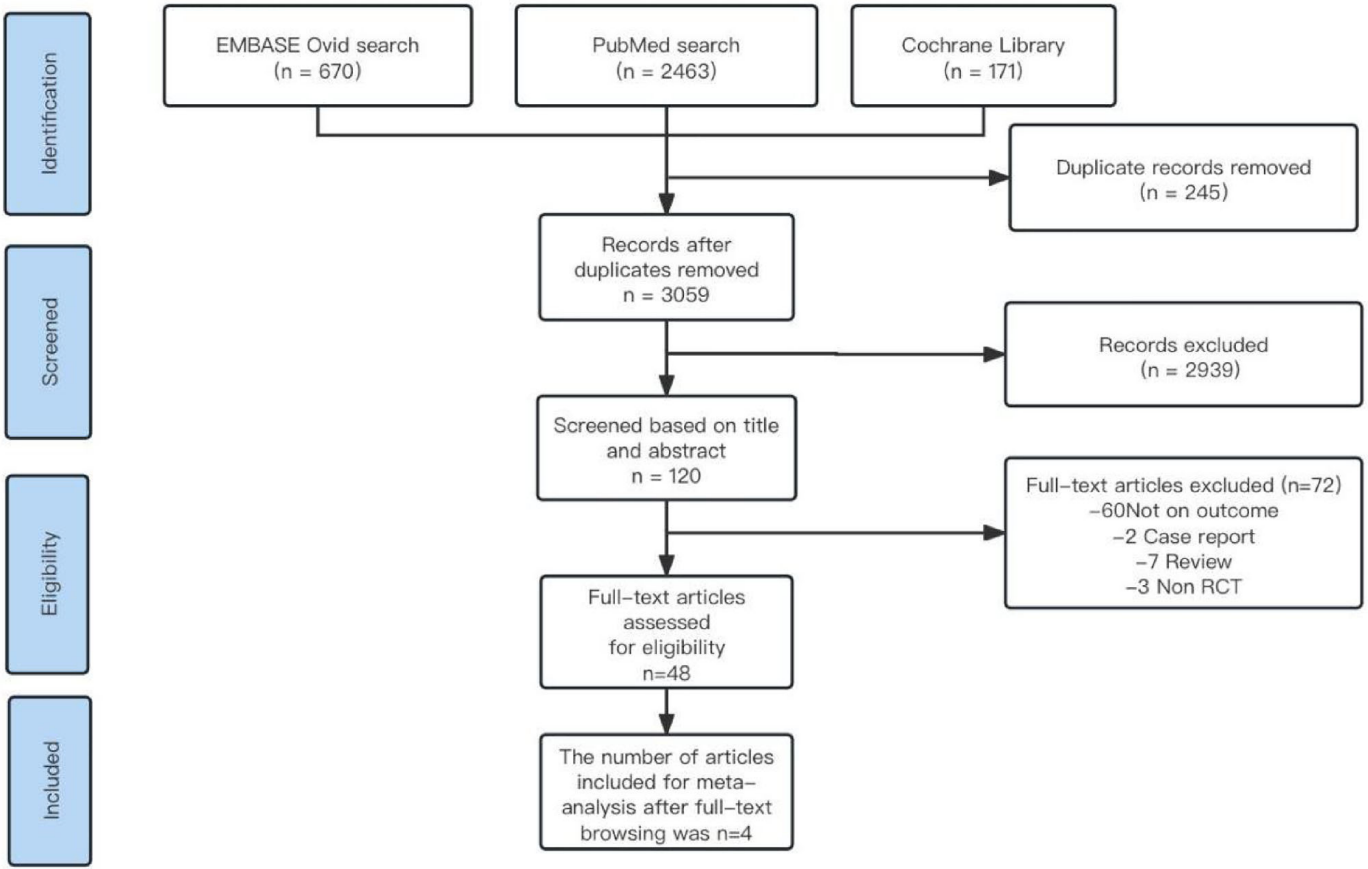
Flowchart of literature search and study selection.

The flowchart in Figure 1 depicts the literature search process. Out of the initially retrieved 3304 articles, 245 were excluded due to duplicate publication, and 2939 were excluded based on titles and abstracts. Among the 48 articles subjected to full‐text assessment, 4 met the inclusion criteria.

### 3.1 Research Characteristics and Quality Assessment

Four randomized controlled trials were included, comprising a total of 1556 study participants. Table 1 summarizes the baseline characteristics of all included studies. The average age of participants was 72 years, with males accounting for 59%.While two studies did not specify the ethnicity of participants, the remaining data indicate a higher proportion of Asian participants in the study cohort. Baseline blood pressure between the intensified treatment and standard treatment groups was similar across included studies. Fifty percent of the studies (2 out of 4) were conducted mainly in European and American countries, while the remaining 50% (2 out of 4) were conducted in Asian countries, all of which were multicenter studies. We obtained additional details for each eligible study meeting the criteria.

**TABLE 1:** Characteristics of the studies included in the meta‐analysis.

				Sex, % (No.)							
Author (year)	Group, No,(Bp range)	Country	Age, mean (SD), year	Female	Male	Medical history, No. (%)	Medications, No. (%)	Favorable clinical outcomes at 90 days No. (%) (mRS score 0–2)	Excellence clinical outcomes at 90 days No. (%) (mRS score 0–1)	Symptomatic ICH No. (%)	Mortality within 90 days No. (%)
Mazighi et al. (2021)	Intensive SBP target group (*n* = 158) (100–129 mmHg)	France	77	48% (143)	52% (175)	Hypertension: 110/157 (70%), diabetes: 34/155 (22%), hypercholesterolemia: 59/153 (39%), current smoking: 19/137 (14%), previous stroke or TIA: 25/156 (16%), previous anti‐thrombotic: 74/156 (47%)	Antiplatelet: 44/156 (28%), anticoagulant: 36/156 (23%)	67/152 (44%)	48/152 (32%)	17/154 (11%)	29/152 (19%)
	Standard SBP target group (*n* = 160) (130–185 mmHg)					Hypertension: 113/160 (71%), diabetes: 33/159 (21%), hypercholesterolemia: 55/158 (35%), current smoking: 24/146 (16%), previous stroke or TIA: 21/159 (13%), previous anti‐thrombotic: 65/160 (41%)	Antiplatelet: 37/160 (23%), Anticoagulant: 34/160 (21%)	69/153 (45%)	42/153 (28%)	11/157 (7%)	21/153 (14%)
Yang et al. (2022)	Intensive SBP target group (*n* = 407). (< 120 mmHg)	China	67 (12)	38%(310)	62%(506)	Hypertension: 267 (66%);previous stroke: 107 (26%);coronary artery disease: 51 (13%);valvular heart disease: 16 (4%);other heart disease: 19 (5%);atrial fibrillation: 84 (21%);diabetes: 81 (20%);hypercholesterolemia: 14 (3%);modified Rankin scale score of 1–2 before stroke onset: 71 (18%)	Antihypertensive drugs: 176 (43%);statin or other lipid‐lowering drug: 30 (7%);aspirin or other antiplatelet drug: 34 (8%);anticoagulation drug: 20 (5%);	192/404 (48%)	154/404 (38%)	23/407 (6%)	66/406 (16%)
	Standard SBP target group (*n* = 409) (140–180 mmHg)					Hypertension: 261 (64%);previous stroke: 139 (34%);coronary artery disease: 59 (14%);valvular heart disease: 17 (4%);other heart disease: 16 (4%);Atrial fibrillation: 98 (24%);diabetes: 82 (20%);hypercholesterolemia: 13 (3%);modified Rankin scale score of 1–2 before stroke onset: 78 (19%)	Antihypertensive drugs: 179 (44%);statin or other lipid‐lowering drug: 30 (7%);aspirin or other antiplatelet drug: 39 (10%);anticoagulation drug: 20 (5%);	247/406 (61%)	191/406 (47%)	25/409 (6%)	61/408 (15%)
Nam et al. (2023)	Intensive SBP target group (*n* = 155) (< 140 mmHg)	South Korea	73 (11.5)	40.4%(121)	59.6 (181)	Hypertension: 121 (78.1%);atrial fibrillation: 77 (49.7%); diabetes: 65 (41.9%);hyperlipidemia: 61 (39.4%); smoking: 39 (25.2%);previous stroke: 36 (23.2%); coronary artery obstructive disease: 18 (11.6%); active cancer: 9 (5.8%); congestive heart failure: 7 (4.5%); peripheral artery occlusive disease: 2 (1.3%)	NA	61/155 (39%)	52/155 (34%)	14/155 (9%)	12/155 (8%)
	Standard SBP target group (*n* = 147) (140‐180 mmHg)					Hypertension: 110 (74.8%);atrial fibrillation: 69 (46.9%); diabetes: 62 (42.2%);hyperlipidemia: 54 (36.7%); smoking: 29 (19.7%);previous stroke: 30 (20.4%); coronary artery obstructive disease: 16 (10.9%); active cancer: 5 (3.4%); congestive heart failure: 7 (4.8%); peripheral artery occlusive disease: 6 (4.1%)	NA	80/147 (54%)	61/147 (41%)	12/149 (8%)	8/147 (5%)
Mistry et al. (2023)	Intensive SBP target group (*n* = 40) (< 140 mmHg)	US	69.6 (14.5)	57.5%(69)	42.5%(51)	Hyperlipidemia: 33.0 (82.5%), hypertension: 32 (80%), atrial fibrillation: 19 (47.5%), diabetes: 12 (30%), current smoking: 8 (20%)	Baseline antihypertensive use: 27 (67.5%), Intravenous thrombolysis: 17 (42.5%), baseline antiplatelet use: 14 (35%), baseline anticoagulant use: 10 (25%)	17/37 (46%)	10/37 (27%)	2/37 (5%)	7/37 (19%)
	Standard SBP target group(*n* = 80) (140‐180 mmHg)					Hyperlipidemia: 28 (70%), hypertension: 28 (70%), atrial fibrillation: 13 (32.5%), diabetes: 15 (37.5%), current smoking: 12 (30%),	Baseline antihypertensive use: 27 (67.5%), intravenous thrombolysis: 18 (45%), baseline antiplatelet use: 19 (47.5%), baseline anticoagulant use: 9 (22.5%)	36/76 (47%)	21/76 (28%)	3/72 (4%)	20/76 (26%)

*Note*: Data are presented as *n* (%) or mean ± standard deviation.

SBP: systolic blood pressure; ICH: intracranial hemorrhage.

### 3.2 The Impact of Intensified Blood Pressure Control on Patient Prognosis

**FIGURE 2:**
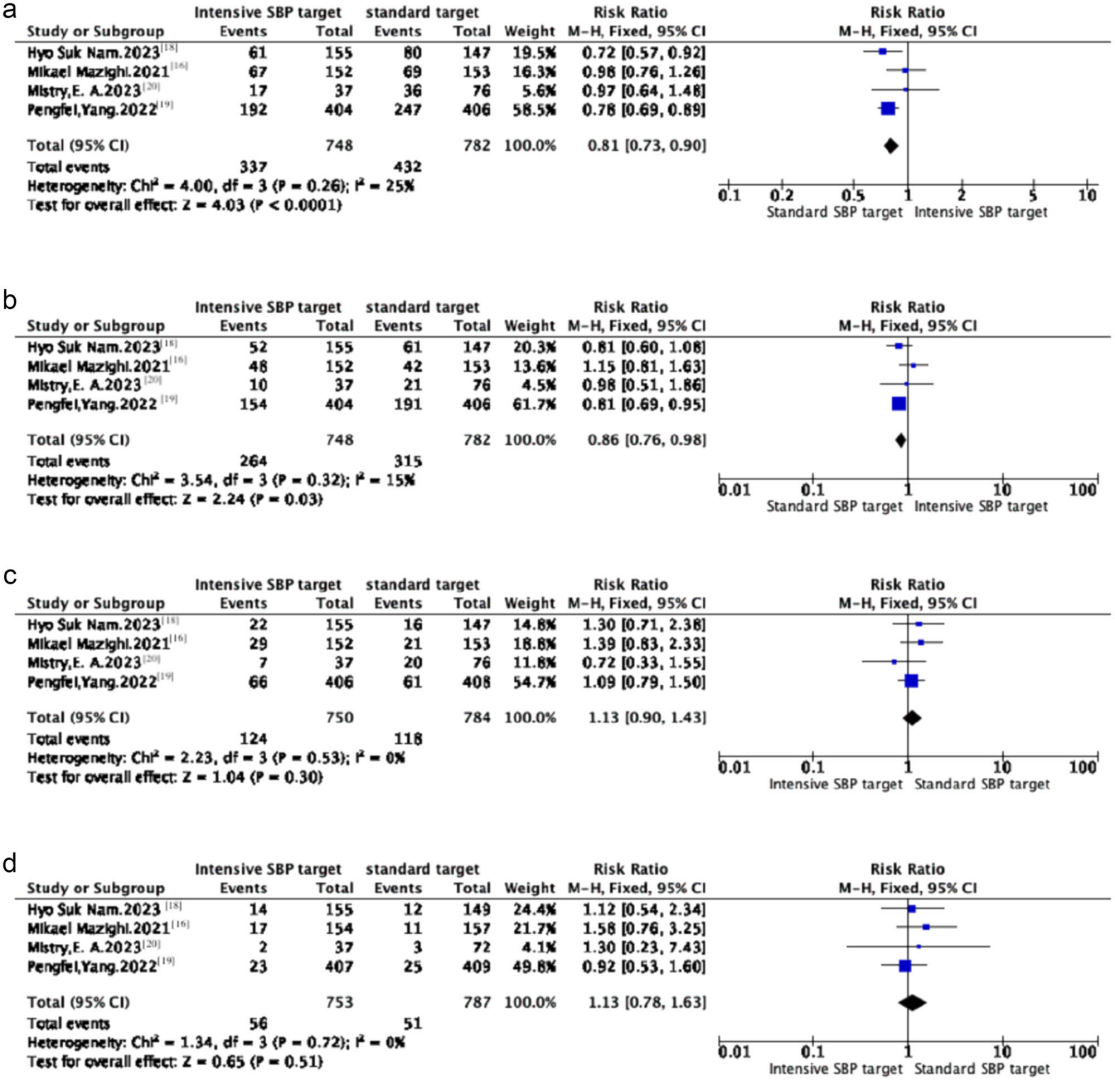
(a) The favorable clinical outcome (mRS ≤ 2) at 90 days between Standard systolic blood pressure (SBP) target and Intensive SBP target. (b) The excellent clinical outcome (mRS ≤ 1) at 90 days between Standard SBP target and Intensive SBP target. (c) The mortality at 90 days of the comparison between Standard SBP target and Intensive SBP target. (d) The occurrence of sICH at 90 days in the comparison between Standard SBP target and Intensive SBP target.

Compared to the intensive blood pressure control group, the standard blood pressure control group demonstrated a higher likelihood of achieving favorable clinical outcomes (mRS ≤ 2). Specifically, in an analysis encompassing four studies, we observed superior clinical outcomes in the standard group (Figure 2), with a RR of 0.81 (95%CI: 0.73–0.90; *p* < 0.05; *I* ^2^  =  25%). Similarly, the standard group demonstrated a higher probability of achieving an excellent clinical outcome (mRS ≤ 1) at 90 days compared to the intensive blood pressure control group. Analysis of data from four studies revealed that the standard group exhibited significantly better clinical outcomes than the intensive blood pressure control group (Figure 2B), with an RR of 0.86 (95% CI: 0.76–0.98; *p* < 0.05; *I* ^2^  =  15%). Moreover, funnel plots indicated no significant publication bias for any outcome, further supporting the reliability of our study findings

### 3.3 Safety Concerns of Intensified Blood Pressure Control

However, upon examining mortality rates, we found no significant difference between the standard and intensive blood pressure control groups at 90 days. Specifically, in an analysis of data from four studies (Figure 2C), the RR of mortality was 1.13 (95% CI: 0.90–1.43; *p*  =  0.30; *I* ^2^  =  0%), indicating no statistically significant difference between the two groups. Similarly, our comparison of safety outcomes related to symptomatic intracranial hemorrhage showed no significant difference. Analysis of data from four studies (Figure 2D) revealed an RR of 1.13 for symptomatic intracranial hemorrhage (95% CI: 0.78–1.63; *p*  =  0.51; *I* ^2^  =  0%), again indicating no statistically significant difference. Therefore, our findings suggest that, regarding safety, there is no significant difference between standard and intensive blood pressure control

## 4 Discussion

In this systematic review and meta‐analysis evaluating blood pressure management strategies following vascular interventions in stroke patients, with patient outcomes assessed over a 3‐month follow‐up period, it was determined that the choice between intensified and standard blood pressure control did not significantly influence patient outcomes. A multicenter retrospective study investigated the effects of different blood pressure targets in patients with acute ischemic stroke following thrombectomy, comparing intensive control (<140 mmHg), moderate control (<160 mmHg), and standard control (<180 mmHg) groups. The study found that intensive blood pressure control was associated with better functional outcomes and fewer complications, such as symptomatic intracranial hemorrhage (Anadani et al. 2020). However, unlike this observational study, intensive blood pressure control did not demonstrate superiority in clinical outcomes over standard control (Mazighi et al. 2021).However, no significant disparity in safety outcomes between the two approaches was observed, including 90‐day mortality and symptomatic intracranial hemorrhage. Notably, there is insufficient evidence to suggest that intensified blood pressure control escalates the risk of adverse events. These estimations exhibit robustness and demonstrate minimal variation in sensitivity analysis.

This review presents the latest insights, incorporating outcome data from 1556 stroke patients postvascular intervention. Four studies were subjected to analysis to assess the efficacy of intensified blood pressure treatment, all of which yielded analogous results. The management of blood pressure poses multifaceted challenges in acute ischemic stroke patients. Swift fluctuations in blood pressure may exacerbate damage to compromised brain tissue, particularly postvascular reperfusion therapy. Furthermore, various factors may impinge upon patients' blood pressure levels, including stroke severity, pretreatment baseline blood pressure levels, and a history of hypertension. Consequently, the formulation of a universally applicable blood pressure management strategy proves exceedingly arduous.

Findings from pertinent nonrandomized controlled clinical trials posit that the adoption of an aggressive blood pressure management strategy augments the recovery of patients successfully undergoing vascular reperfusion therapy. Specifically, some trials suggest that setting a systolic blood pressure target below 140 mm Hg engenders relatively superior functional outcomes compared to traditional management modalities (Anadani et al. 2019; Mistry et al. 2018; Maïer et al. 2020; Chang and Han 2019). Nonetheless, recent meta‐analyses of randomized controlled trials proffer contrary conclusions. Intensified blood pressure control, particularly with a systolic blood pressure target of 140 mm Hg, despite evincing no elevated rates of symptomatic intracranial hemorrhage or mortality, engenders diminished functional recovery in patients. It is important to note that the experimental design of randomized controlled trials (RCTs) may influence the effectiveness of interventions. In the standard treatment group, patients with blood pressure below 140 mmHg did not receive interventions to raise their blood pressure. This means that some patients did not actually receive the intended intervention, which may have diluted the observed treatment effect. This design differs from the clinical practice in observational studies and may be one of the reasons for the discrepancies between the results of RCTs and observational studies. Future research should consider this factor and optimize experimental designs to more accurately assess the effects of blood pressure management strategies. It is postulated that excessive blood pressure reduction during control may precipitate inadequate vascular perfusion, exacerbating ischemia (Nam et al. 2023). Moreover, patients evincing poor vascular elasticity may exacerbate this phenomenon. Elevated rates of malignant brain edema occurrence have been observed in some studies, with several trials prematurely halted due to significantly inferior 90‐day outcomes in the intensified blood pressure control cohort (Yang et al. 2022). Recent investigations have further contended that both excessively high and low blood pressure may engender divergent impacts (Mistry et al. 2023). Consequently, this study undertook a nuanced subdivision of blood pressure control into three cohorts: <140 mm Hg, <160 mm Hg, <180 mm Hg. Nevertheless, even within the intensified blood pressure control <160 mm Hg subgroup, no discernibly superior outcomes were discerned. Given that elevated blood pressure constitutes a self‐regulatory mechanism in response to exacerbated edema, which compresses blood vessels and exacerbates cerebral ischemia, the body initiates perfusion pressure augmentation through self‐regulation. (Lattanzi et al. 2013; Alqadri, Sreenivasan, and Qureshi 2013). However, excessive blood pressure elevation escalates the risk of complications, underscoring the imperative to ascertain an optimal equilibrium.

Despite the discordance in current clinical trial findings, they proffer valuable insights for prospective research endeavors. Further elucidation is warranted concerning the effects of diverse blood pressure management strategies in specific patient subgroups, necessitating stratified trials predicated on disparate baseline blood pressure levels. Patients evincing diverse baseline levels may manifest disparate vascular elasticity and edema severity, necessitating augmented perfusion pressure to sustain normative cerebral perfusion. Furthermore, patients undergoing specific treatments, such as edema control and multifarious complication management, warrant tailored intervention to engender enhanced clinical outcomes. A potential research direction is to stratify patients after mechanical reperfusion therapy based on their blood pressure levels for tailored management. For patients with lower blood pressure (<140 mmHg), nonpharmacological methods could be employed to raise blood pressure to <160 mmHg to assess whether increasing blood pressure improves perfusion and functional outcomes. Meanwhile, another group could maintain their current blood pressure levels, and a third group could have their blood pressure further reduced to <130 mmHg to observe the impact of additional blood pressure reduction. For postoperative patients with higher blood pressure (140–180 mmHg), they could be randomly assigned to a blood pressure reduction group or a nonintervention group to compare the effects of these strategies on patient outcomes. Such a design would enable us to more precisely determine the optimal blood pressure management targets for patients with different baseline blood pressure levels, thereby achieving more individualized treatment approaches. Subsequently, expansive‐scale and protracted follow‐up studies are imperative to adjudicate the long‐term ramifications of diverse blood pressure management strategies on patient outcomes. Moreover, predicated on clinical experience and evolving methodologies, extant blood pressure management guidelines necessitate refinement and updating to perpetuate enhanced clinical practice.

Blood pressure management in acute ischemic stroke patients represents a multifaceted and pivotal concern. Recent clinical trial outcomes intimate that unduly reducing blood pressure may prove deleterious to recovery and exacerbate the risk of sundry complications in patients successfully undergoing vascular reperfusion therapy. Hence, personalized blood pressure management strategies are indispensable, necessitating individualized adjustments contingent upon patient‐specific circumstances and treatment backgrounds. Subsequent research endeavors should prioritize the delineation of the long‐term effects of diverse blood pressure management strategies and the refinement and updating of pertinent clinical guidelines to ameliorate patient recovery and prognosis.

### 4.1 Limitations

Firstly, the included RCTs exhibit significant differences in participants' demographic characteristics, baseline characteristics, and inclusion criteria. This variability may introduce heterogeneity, affecting the generalizability of the results. Variations in stroke severity, comorbid conditions, and treatment protocols across studies may impact outcomes and complicate direct comparisons. Secondly, this meta‐analysis includes only four studies with a total of 753 patients. The relatively small sample size may limit the statistical power of the analysis, potentially obscuring smaller but clinically significant differences between intensive and standard blood pressure control strategies. Thirdly, the targets for intensive blood pressure control in the included studies are not uniform. Some studies aimed for a SBP of <140 mm Hg, while others had different thresholds. This lack of standardization makes it difficult to determine the optimal blood pressure management target after EVT. Moreover, the analysis did not include unpublished studies, which could lead to publication bias. Studies with negative or null results are less likely to be published, potentially skewing the meta‐analysis toward positive outcomes. Finally, only English‐language studies were included, excluding non‐English literature. This may result in language bias and the omission of relevant data published in other languages.

## 5 Conclusion

After careful analysis, our conclusion is that intensified blood pressure control compared to standard blood pressure control following endovascular treatment in acute stroke patients does not yield better clinical outcomes and may even lead to inferior ones. Moreover, there is no significant disparity in terms of safety between the two approaches. However, due to the limited number of studies and potential variations in patient populations and methodologies, further analysis is warranted. We encourage additional large‐scale, high‐quality randomized controlled trials to confirm these findings and to establish optimal blood pressure management strategies for this patient population

## Author Contributions

**Qiang Ji Bao**: Conceptualization, methodology, software, writing—original draft, validation, formal analysis, visualization. **Yi Ming Li**: Conceptualization, methodology, software, writing—original draft, validation, visualization, formal analysis. **Xinting Wu**: Data curation, investigation, software. **Yun Ting Li**: Software, data curation, investigation. **Xiao Long Huang**: Software, data curation, investigation. **Hui Zhou**: Software, data curation, investigation. **Xiao Qiang Zhang**: Writing—review and editing, supervision, resources, project administration, methodology, funding acquisition. **Xue Jun Wang**: Supervision, writing—review and editing, resources, project administration, methodology, funding acquisition.

## Ethics Statement

An ethics statement was not required for this study type; no human or animal subjects or materials were used.

## Conflicts of Interest

The authors declare that there is no conflict of interest regarding the publication of this article.

### Peer Review

The peer review history for this article is available at https://publons.com/publon/10.1002/brb3.70211

## Supporting information



Figure S1. mRS ≤ 1 at 90 days.
Figure S2. mRS ≤ 2 at 90 days.
Figure S3. 90‐day mortality.
Figure S4. Symptomatic intracranial bleeding.

## Data Availability

All data generated or analyzed during this study are included in this article and its online supplementary material files. Further inquiries can be directed to the corresponding author.
