# Supplementary material for: Meta‐Analysis of the Safety and Efficacy of Intensive Blood Pressure Control After Thrombectomy
Source: Brain Behav. 2025 Feb 9;15(2):e70211. doi: 10.1002/brb3.70211 (PMC11807842; doi:10.1002/brb3.70211)

Supplementary Materials:Funnel Plots

Figure S1.mRS≤1 at 90 days


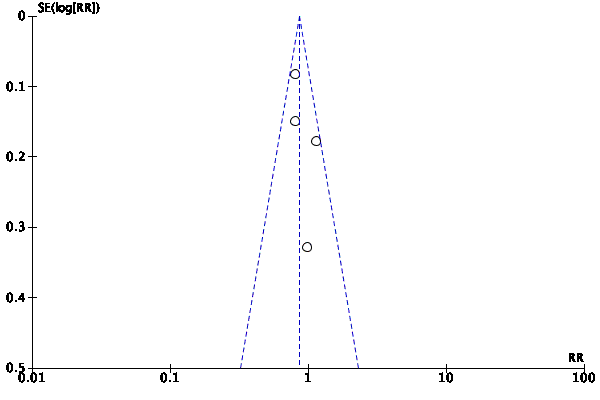


Figure S2.mRS≤2 at 90 days


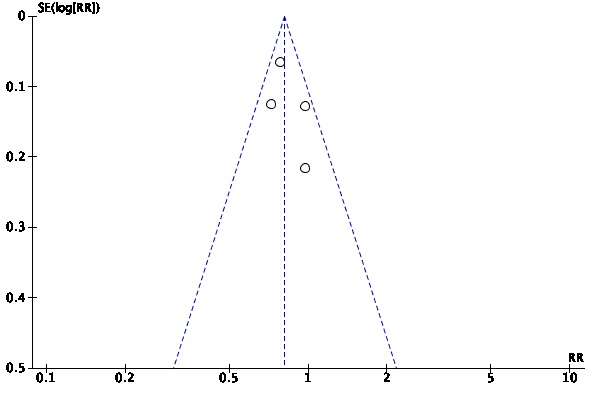


Figure S3.90 day mortality


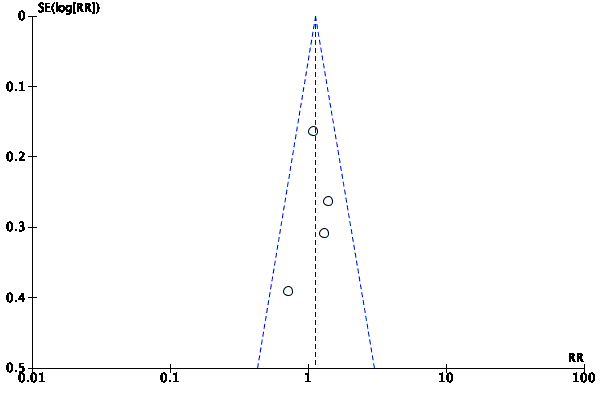


Figure S4.Symptomatic intracranial bleeding


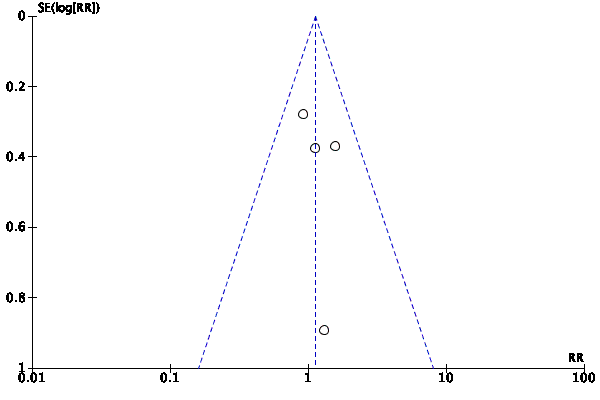

Supplement: Supplementary file 1 — Figure S1. mRS ≤ 1 at 90 days. Figure S2. mRS ≤ 2 at 90 days. Figure S3. 90‐day mortality. Figure S4. Symptomatic intracranial bleeding. [file BRB3-15-e70211-s001.docx]
